# Supplementary material for: The interactome of intact mitochondria by cross-linking mass spectrometry provides evidence for coexisting respiratory supercomplexes
Source: Mol Cell Proteomics. 2017 Dec 8;17(2):216–32. doi: 10.1074/mcp.RA117.000470 (PMC5795388; doi:10.1074/mcp.RA117.000470)
Supplement: Supplemental Data [file supp_RA117.000470_133922_0_supp_23978_fzffwf.zip › spectra_annotation/mito_spectra_annotation/53-1-6-1-3-1.pdf]

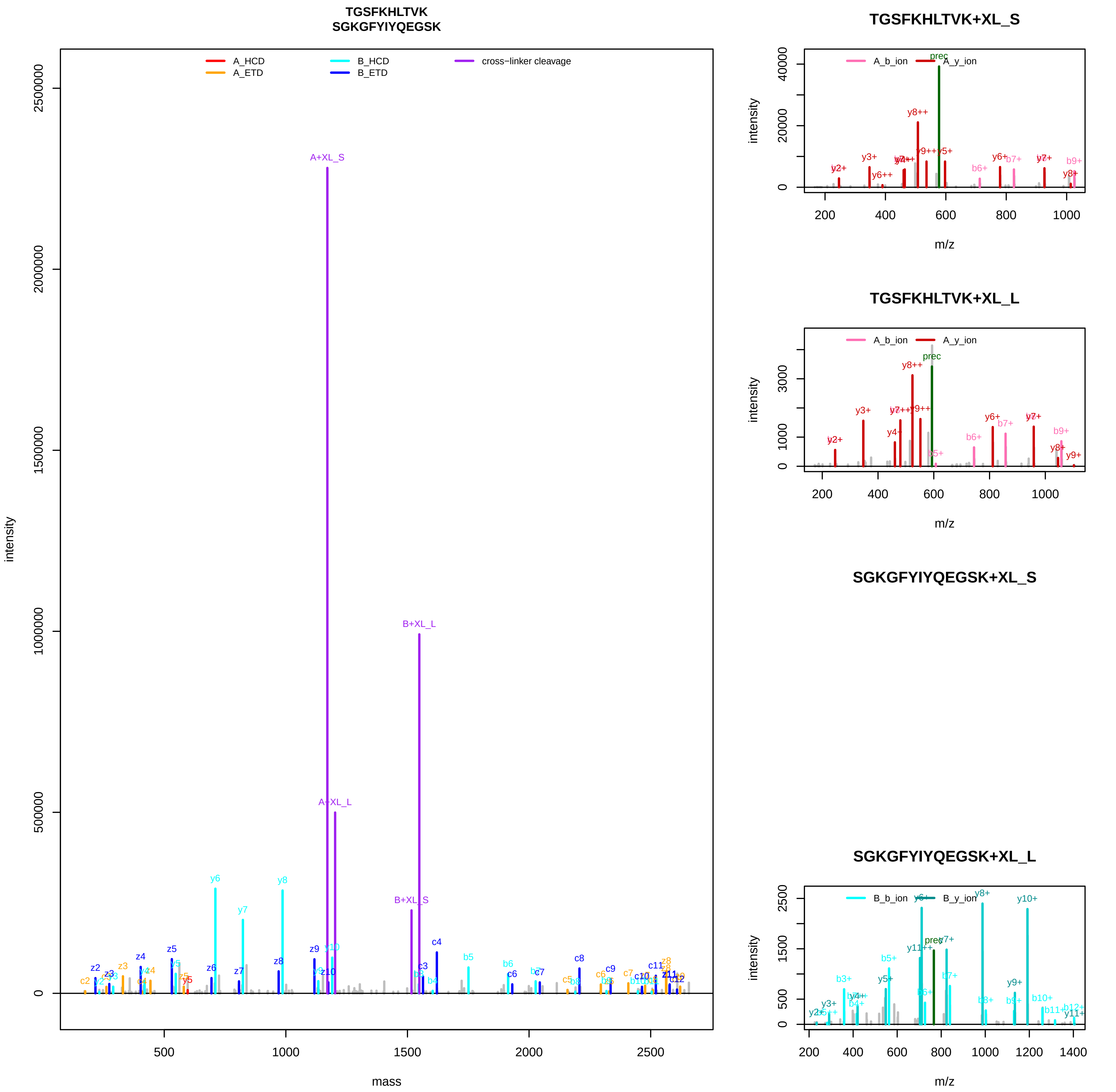
TGSFKHLTVK
SGKGFYIYQEGSK
A_HCD
A_ETD
B_HCD
B_ETD
cross−linker cleavage
A+XL_S
B+XL_L
A+XL_L
B+XL_S
y6
y8
y7
y10
z9
z8
z7
z6
z5
z4
z3
z2
c2
c4
c3
c6
c7
c8
c9
c10
c11
c12
b5
b6
z10
intensity
mass
0
500000
1000000
1500000
2000000
2500000
500
1000
1500
2000
2500
TGSFKHLTVK+XL_S
A_b_ion
A_y_ion
prec
y8++
y9++
y5+
y3+
y6++
y2+
y6+
b6+
b7+
y7+
b9+
y8+
intensity
m/z
0
20000
40000
200
400
600
800
1000
TGSFKHLTVK+XL_L
A_b_ion
A_y_ion
prec
y8++
y9++
y3+
y4+
y2+
b5+
b6+
y6+
b7+
y7+
b9+
y8+
y9+
intensity
m/z
0
1000
2000
3000
200
400
600
800
1000
SGKGFYIYQEGSK+XL_S
SGKGFYIYQEGSK+XL_L
B_b_ion
B_y_ion
y6+
y8+
y10+
prec
y7+
y11++
b5+
y5+
b3+
y3+
y4+
b4+
b6+
b7+
y9+
b8+
b9+
b10+
b11+
b12+
y11+
y2+
intensity
m/z
0
500
1500
2500
200
400
600
800
1000
1200
1400
